# A Novel Cancer Vaccine Strategy Based on HLA-A*0201 Matched Allogeneic Plasmacytoid Dendritic Cells

**DOI:** 10.1371/journal.pone.0010458

**Published:** 2010-05-04

**Authors:** Caroline Aspord, Julie Charles, Marie-Therese Leccia, David Laurin, Marie-Jeanne Richard, Laurence Chaperot, Joel Plumas

**Affiliations:** 1 Etablissement Français du Sang Rhone-Alpes, R&D Laboratory, La Tronche, France; 2 University Joseph Fourier, Grenoble, France; 3 INSERM, U823, Immunobiology & Immunotherapy of Cancers, La Tronche, France; 4 Centre Hospitalier Universitaire Grenoble, Michallon Hospital, Dermatology, pole pluridisciplinaire de medecine, Grenoble, France; 5 Centre Hospitalier Universitaire Grenoble, Michallon Hospital, Cancerology and Biotherapy, Grenoble, France; University of Sheffield, United Kingdom

## Abstract

**Background:**

The development of effective cancer vaccines still remains a challenge. Despite the crucial role of plasmacytoid dendritic cells (pDCs) in anti-tumor responses, their therapeutic potential has not yet been worked out. We explored the relevance of HLA-A*0201 matched allogeneic pDCs as vectors for immunotherapy.

**Methods and Findings:**

Stimulation of PBMC from HLA-A*0201^+^ donors by HLA-A*0201 matched allogeneic pDCs pulsed with tumor-derived peptides triggered high levels of antigen-specific and functional cytotoxic T cell responses (up to 98% tetramer^+^ CD8 T cells). The pDC vaccine demonstrated strong anti-tumor therapeutic in vivo efficacy as shown by the inhibition of tumor growth in a humanized mouse model. It also elicited highly functional tumor-specific T cells ex-vivo from PBMC and TIL of stage I-IV melanoma patients. Responses against MelA, GP100, tyrosinase and MAGE-3 antigens reached tetramer levels up to 62%, 24%, 85% and 4.3% respectively. pDC vaccine-primed T cells specifically killed patients' own autologous melanoma tumor cells. This semi-allogeneic pDC vaccine was more effective than conventional myeloid DC-based vaccines. Furthermore, the pDC vaccine design endows it with a strong potential for clinical application in cancer treatment.

**Conclusions:**

These findings highlight HLA-A*0201 matched allogeneic pDCs as potent inducers of tumor immunity and provide a promising immunotherapeutic strategy to fight cancer.

## Introduction

The development of effective vaccines for cancer treatment represents a major public health issue [1]. Because cytotoxic T lymphocytes (CTL) are able to recognize and lyse malignant cells, many therapeutic trials have been designed to potentiate CTL responses. Myeloid dendritic cells (mDC)-based vaccines succeeded in inducing specific T cells in patients but without sufficient clinical efficacy [2], [3]. Adoptive cellular transfer of anti-tumor effector T cells amplified ex-vivo from TIL induced objective tumor regression [4], [5], but the complexity of this strategy has hindered wide development. Therefore, there is a strong need for novel immunotherapeutic strategies to overcome the limitations of current protocols.

Up to now, the induction of specific T cell responses for both adoptive and active immunotherapeutic strategies has been based on mDCs [6]–[8]. Plasmacytoid dendritic cells (pDC) are however key players in immunity [9], [10] with a role in tumor-specific immune responses [11]. pDCs differ from mDCs in many aspects such as TLR expression, migration profile and immune responses triggering. pDC are also capable of antigen capture, processing and presentation [12]–[15]. Antigen-pulsed pDC can stimulate specific primary (MelA) and memory (Flu) autologous CD4 and CD8 T cell immune responses in vitro [16]–[19] and prime functional T cell responses in vivo as shown after vaccination of mice with CpG or virus-activated pDC [20]–[21]. pDC are found within many tumors in humans [22]–[26], where they are thought to be immature, tolerogenic or associated with poor prognosis. However, in melanoma, pDC activation by TLR-L could trigger potent anti-tumor effects. In mice, imiquimod application (TLR7-L) [27] or intratumoral injection of CpG (TLR9-L) [28] reversed the functional inhibition of pDC, thereby promoting tumor regression. Moreover local CpG administration in melanoma patients induced the recruitment and activation of pDC in sentinel lymph nodes [29] and subsequent tumor-specific CD8 T cells associated with clinical benefit [30]. The potential of pDC in generating effective tumor-specific immune responses has also been demonstrated in a mouse model [31]. pDC-based approaches and TLR agonists [32] are therefore promising for the treatment of human cancer.

Tumor antigens usually trigger weak responses. In contrast, allogeneic responses directed against non-self MHC are extremely potent. Interestingly, the allogeneic response mediated by MHC class II-restricted CD4+ T cells promotes bystander specific T cell induction [33], [34] as already shown with viral peptides [35] and tumor regression following allogeneic skin graft [36]. Allogeneicity could therefore be exploited to promote immunogenicity towards tumor antigens [37] when considering a partial HLA match between the vaccine and the patient, further referred to as HLA matched allogeneicity.

Because pDCs play a fundamental role in triggering T cell responses, their use could be promising as new immunotherapeutic strategies. However, the use of autologous pDC for cancer immunotherapy is difficult because of the scarcity of these cells [38] and the possible functional alteration of pDCs harvested from tumor-bearing patients. We therefore explored the potential of HLA-A*0201 matched allogeneic pDC to induce HLA-A*0201-restricted anti-tumor immunity. We used a unique human pDC cell line (GEN) established from leukaemic HLA-A*0201^+^ pDC with phenotypic and functional features closed to primary pDCs [39], [40], [41]. The strategy consisted of using the peptide-loaded pDCs to induce HLA-A*0201-restricted antigen-specific CTL. We demonstrate here using tumor and viral model antigens the potential of the irradiated peptide-pulsed human HLA-A*0201 matched allogeneic pDC line (GENiusVac) in vitro, its therapeutic efficacy in vivo in humanized mice, and its clinical relevance ex-vivo with melanoma patients' cells. Our findings highlight HLA-A*0201 matched allogeneic pDCs as potent inducers of anti-tumor immunity and provide a promising new immunotherapeutic strategy to fight cancer.

## Materials and Methods

### Cell lines and reagents

Cultures were performed in RPMI 1640 Glutamax supplemented with 1% non-essential amino acids, 1 mM sodium pyruvate (Sigma), 100 µg/ml gentamycin and 10% FCS (all from Invitrogen unless notified). Melanoma line Me275 was provided by Pr J-C Cerottini (Ludwig Institute for Cancer Research, Epalinges, Switzerland). Melanoma lines COLO829 and A375, T2 and K562 lines were purchased from ATCC (LGC Standards, Molsheim, France). Melanoma line Mel89 was generated in our laboratory (Figure S1). Anti-human CD45, CD3, CD8 Abs were purchased from Beckman Coulter. Anti-HLA-A2 Abs were purchased from BD Biosciences and anti- human MelA from Sigma.

### Peptides and tetramers

We used the following viral- and tumor-derived HLA-A*0201 restricted peptides (NeoMPS) and the corresponding iTag HLA-A*0201 tetramers (Beckman Immunomics): MelA_26–35_ (ELAGIGILTV), GP100_209–217_ (IMDQVPFSV), tyrosinase_369–377_ (YMDGTMSQV), MAGE-3_271–279_ (FLWGPRALV), FluM1_58–66_ (GILGFVFTL), CMVpp65_495–503_ (NLVPMVATV).

### PBMC, pDC line, primary pDC isolation and mDCs generation

Human PBMC were obtained from HLA-A*0201^+^ healthy donors by Ficoll-Hypaque density gradient centrifugation (Eurobio). The human pDC line GEN2.2 was cultured as previously described [39]. Primary pDC were isolated from the blood of HLA-A*0201^+^ healthy donors. DCs were first enriched by depletion of unwanted cells using the Pan DC pre-enrichment kit (StemCell). Recovered cells were either submitted to BDCA4+ selection (Miltenyi) or labelled with a Lineage cocktail, CD123, HLA-DR and CD11c antibodies (BD) and sorted on a BD FACSAria on the basis of CD123 and HLA-DR expression and lack of Lin and CD11c markers. The purity of pDC after sort was over 98%. mDCs were differentiated from blood monocytes using 500 U/ml GM-CSF and 10 ng/ml IL-4 (TEBU Prepotech, France) for 6 days. This study was conducted under a procedure approved by the French Blood Agency Institutional Review Board. All donors signed informed consent forms.

### Melanoma patient samples

Samples were obtained from stage I to IV HLA-A*0201 melanoma patients who signed informed consent forms. We used extra material that was not required for patients' diagnosis or analysis and didn't required supplementary procedures. Therefore in accordance with the French regulation, no ethic approval was required but information and signed consent of the patients. Clinical parameters are shown in Table S1. Blood samples were obtained from 12 patients and PBMC purified by gradient density. Fresh tumor samples were obtained from 6 patients who underwent surgery for in-transit metastasis. Samples were dilacerated and digested in 2 mg/ml collagenase D (Roche Diagnostics) 20 U/ml DNase (Sigma). Then tumor cells were isolated from the cell suspension by adherence and TIL enriched from the non-adherent fraction by density gradient.

### Specific T cell response induction in vitro

GEN cells were first loaded with one or several peptide(s) of interest. Briefly, cells were washed 3 times with serum-free RPMI and resuspended at 1.10^6^ cells/ml. β2-microglobulin (0.1 µg/ml final) (Sigma) and peptide(s) (1–10 µM final) (NeoMPS) were added. After 3 hours of incubation at 37°C, cells were washed twice, irradiated with 30Gy and resuspended at 2.10^5^ cells/ml in RPMI with 10% FCS. Peptide-loaded GEN were then co-cultured with semi-allogeneic HLA-A*0201 PBMC at a 1∶10 ratio in RPMI + 10% FCS for at least 7 days. Cultures were weekly restimulated with peptide-loaded GEN and 200 U/ml IL-2 (Proleukine; Chiron). In some experiments, unstimulated primary pDCs or mDCs matured with LPS (1 µg/ml) were used following the same conditions. In some experiments blocking anti-IFNα (50.000 U/ml) and anti-IFNβ (25.000 U/ml) antibodies (PBL Medical laboratories) or control goat IgG were added at day 0 and day 2 of culture. Specific CD8 T cell responses were measured by tetramer labelling of PBMC initially and at different steps of the culture. Cells were resuspended in HBSS with 2% FCS, stained with CD45 FITC, iTAg HLA-A*0201 tetramer PE, CD3 PC7, CD8 APC antibodies and analyzed by flow cytometry analysis using a FACSCalibur and Cell Quest software (Becton Dickinson). To determine initial tetramer levels, at least 1–2.10^6^ events were acquired.

### In vitro functional assays

#### IFNγ secretion and CD107 expression by tetramer+ CD8 T cells

T cells were first labelled with iTAg HLA-A*0201 tetramer PE for 30 min at RT, washed and restimulated with peptide-pulsed T2 cells (10∶1 ratio) for 5 h30. For IFNγ secretion, 1 µl/ml brefeldin A (BD Biosciences) was added for the last 3 h. Cells were then surface-labelled with anti-CD3 PC7 and anti-CD8 APC antibodies and submitted to IFNγ intracellular staining (BD Biosciences). For CD107 detection, anti-CD107a and CD107b FITC antibodies (10 µl/1.10^6^ cells) (BD Biosciences) were added in the medium at the beginning of the restimulation in presence of Golgi STOP (0.67 µl/ml) for the last 4 h. Cells were then labelled with anti-CD3 PC7 and anti-CD8 APC antibodies. IFNγ and CD107 staining were analyzed on the tetramer^+^ CD8+ T cells, tetramer^-^ CD8+ T cells and CD4+ T cells.

#### Cytotoxicity assay

Antigen-specific cytotoxic activity was measured by performing a standard ^51^Cr release assay. Effector T cells were sorted from the co-culture using an EasySep human T cell enrichment kit (Stem Cell). Target cells (peptide-pulsed T2 cells, K562, allogeneic or autologous tumor cells) were labeled with 50 µCi for 1 hour at 37°C, washed 3 times and plated with effector T cells at the indicated E:T ratio in round bottom 96-well plates. After 4 hours of incubation, radioactivity was measured on 30 µl of supernatants on a microplate scintillation counter Top Count NXT (Perkin Elmer). The mean of triplicate measurements was expressed as a percentage of specific lysis using the formula: (sample release – spontaneous release)/(maximal release – spontaneous release) ×100.

### In vivo functional assays in humanized mice

NOD-SCID β_2_m^-/-^ immunodeficient mice (NOD.Cg-Prkdc^SCID^β_2_m^Tm1Unc^/J) were purchased from Jackson ImmunoResearch Laboratories (Bar Harbor, USA) and bred at the Plateforme de Haute Technologie Animale (PHTA, La Tronche, France). For active therapy experiments, HuPBL mice were constructed by transplanting intraperitoneally (ip) 50.10^6^ PBMC from healthy donors into sublethally irradiated NOD-SCID β_2_m^-/-^ mice (120–150 cGy). Mice were further vaccinated with 5.10^6^ irradiated peptide-pulsed GEN cells by ip or sc routes once a week. Response to vaccination was analysed at different time points in blood, peritoneal lavage, spleen and lymph nodes. Organs were digested 30 min at 37°C with 2 mg/ml collagenase D (Roche Diagnostics). Resultant cell suspensions were washed with HBSS + 2% FCS, stained using the following anti-human antibodies (CD45 FITC, iTAg HLA-A*0201 tetramer PE, CD3 PC7, CD8 APC) and submitted to flow cytometry analysis. To assess therapeutic efficiency, 1.10^6^ human tumor cells were implanted subcutaneously into the flank of the humanized mice either 5 days after (prophylactic) or 4 days before (therapeutic) the first vaccination. Vaccination was repeated every week. Tumor size was monitored every 2–3 days and tumor volume calculated using the formula: (short diameter)^2^× long diameter/2. To analyse specific T cells at the tumor site and in DLN, tissues were digested as previously described and cell suspensions were submitted to tetramer labelling and flow cytometry analysis. All in-vivo experiments have been approved by the Regional Committee for Animal Ethic Rhone-Alpes (CREEA) affiliated to the CNRS.

### Statistical analysis

The statistical analyses were performed by using Mann-Whitney non parametric U test and unpaired t test using Prism software.

## Results

### Human HLA matched allogeneic pDCs induce antigen-specific T cell responses from healthy donor PBMC with a strong efficacy in vitro

**Figure 1:**
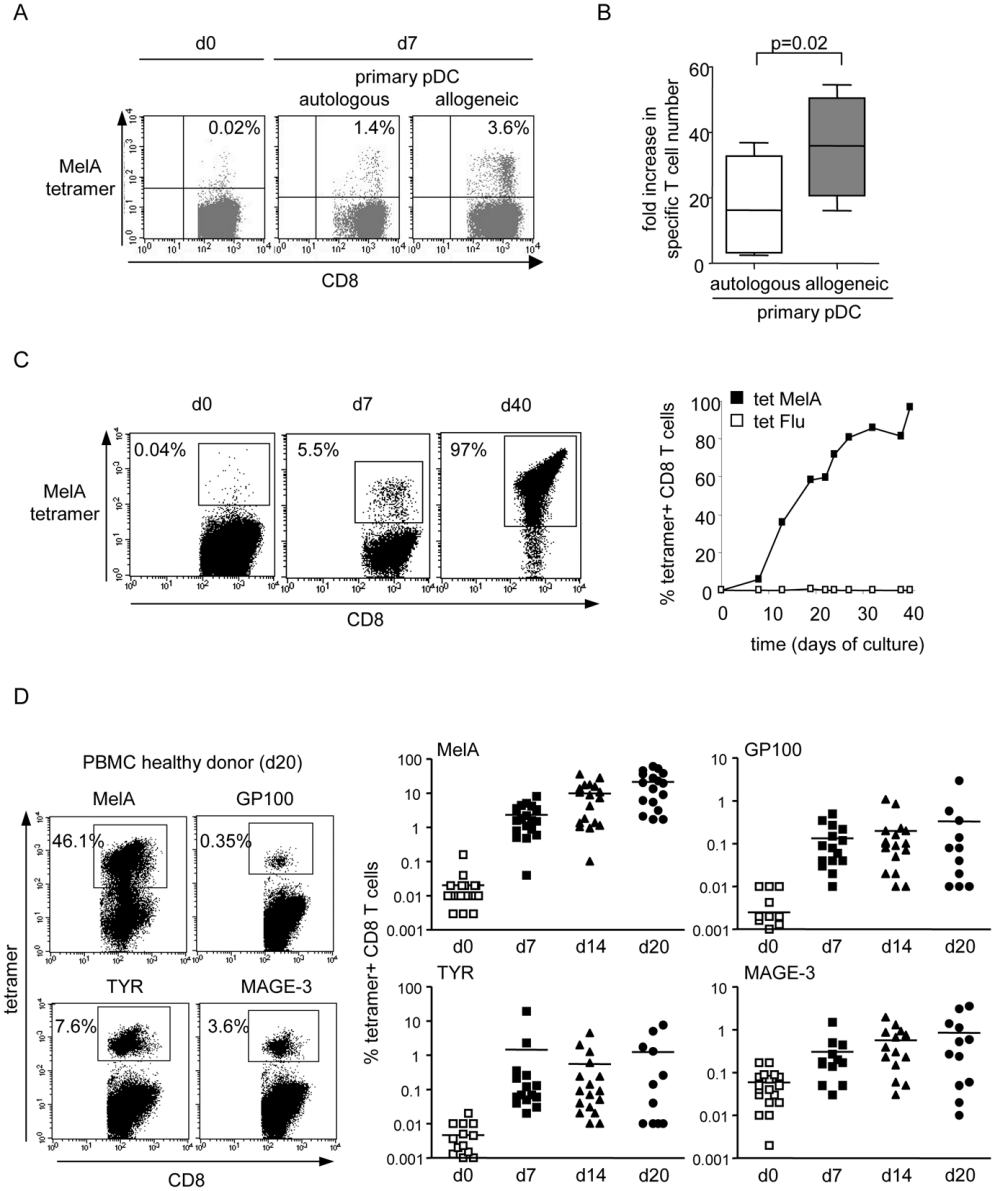
HLA-A*0201 matched allogeneic pDCs induce highly effective tumor-specific T cell responses from HLA-A*0201^+^ healthy donors' PBMC in vitro. (A,B) Autologous or allogeneic HLA-A*0201^+^ primary pDC sorted from the blood of healthy donors were pulsed with MelA peptide and used to stimulate HLA-A*0201^+^ PBMC. The specific T cell response was analyzed at d7 by tetramer labelling. (A) Percentage of MelA specific T cells (gated on CD8+ T cells). One representative experiment is shown. (B) Amplification of the absolute number of specific T cells from d0 to d7 (4 independent experiments performed with 3 different donors). (C,D) allogeneic HLA-A*0201^+^ PBMC from healthy donors were stimulated with the irradiated peptide-loaded HLA-A*0201^+^ pDC line and weekly restimulated in the presence of IL2. Specificity of the T cells was determined by tetramer labelling and flow cytometry analysis. (C) Representative dotplots gated on CD8+ T cells (left panel) and percentages (right panel) of MelA tetramer^+^ T cells in the culture initially and at different time points after stimulation with the pDC line loaded with MelA peptide. Flu tetramer was used as control. (D) Representative dot plots gated on CD8+ T cells (left panel) and percentages of tetramer^+^ CD8+ T cells obtained at days 7, 14 and 20 of the culture towards MelA (n = 18), GP100 (n = 16), TYR (n = 16) and MAGE-3 (n = 16) tumor antigens.

To investigate the potential of HLA matched allogeneic pDC in antigen-specific T cell responses induction, we compared the ability of peptide-loaded primary pDC sorted from healthy donors' blood to induce specific T cell responses in autologous and allogeneic HLA-A*0201-matched settings. pDC led to a significantly higher specific T cell induction in HLA-A*0201 matched allogeneic settings compared to autologous conditions (amplification of the absolute number of MelA-specific T cells in 7 days: 35.6±8.9 vs 17.9±8.7, mean±SEM, p = 0.02) (Figure 1A and 1B, four experiments performed with three different donors). As the scarcity of primary pDC prevents their wide therapeutic use, we used the human pDC cell line (GEN) established from leukaemic HLA-A*0201^+^ pDC as a source of HLA-A*0201 matched allogeneic pDCs. To assess whether the irradiated HLA-A*0201^+^ pDC line can induce specific T cell responses like primary pDC in vitro, allogeneic HLA-A*0201^+^ PBMC from healthy donors were stimulated with the irradiated peptide-loaded GEN cells. For both tumor (MelA) and viral (Flu)-derived peptides, we obtained a massive amplification of specific T cells after only 7 days of culture as detected by tetramer labelling (Figure 1C, Figure S2). This induction was further enhanced by serial stimulations every 7 days with the peptide-loaded pDC line, in combination with IL-2. We routinely obtained 5–25% of tetramer^+^ CD8 T cells after 7 days, 40–60% after 15 days, and up to 98% after 40 days (Figure 1C). Such high responses were reproduced with cells from 14–20 healthy donors and with various melanoma tumor-derived antigens such as MelA, GP100, TYR, MAGE-3 (Figure 1D) as well as virus-derived antigens (Figure S2). Tumor-specific tetramer^+^ T cell responses reached averages of 22% for MelA (range 2–60%), 0.3% for GP100 (range 0–3%), 1.2% for TYR (range 0–8%) and 0.84% for MAGE-3 (range 0–4%) in 20 days. Multi-specific responses were also induced using GEN cells loaded with several different peptides (not shown). Thus, HLA matched allogeneic primary pDCs or the pDC line elicit strong primary and memory antigen-specific T cell responses in vitro from healthy donors.

### The specific T cells induced by HLA matched allogeneic pDC exhibited in vitro functional HLA-restricted activity

**Figure 2:**
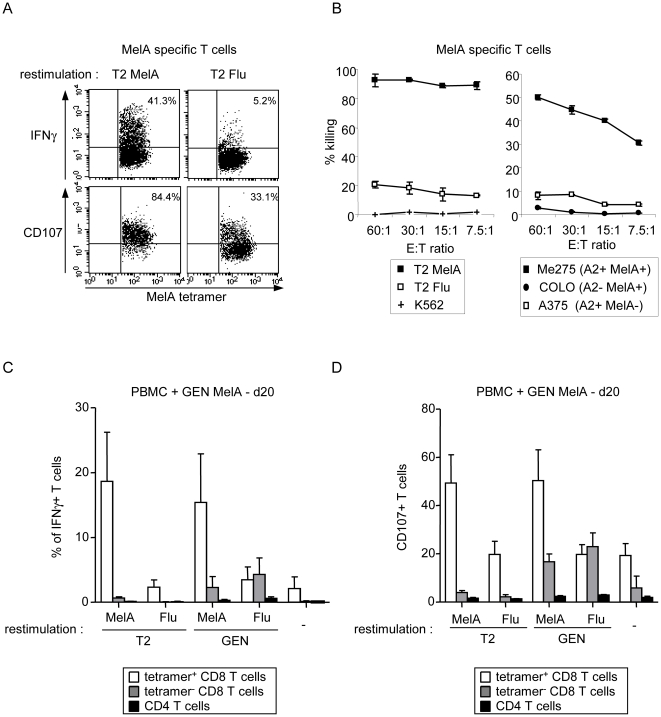
The tumor-specific T cells primed by the HLA-A*0201 matched allogeneic pDC line in vitro exhibited functional antigen- and HLA-A*0201-specific activity. (A) MelA-specific T cells induced by the pDC line secrete IFNγ and express CD107 on the surface upon specific restimulation. Cells from the culture (day 14) were submitted to tetramer labelling and restimulated with T2 cells pulsed with a relevant or control peptide. IFNγ production was assessed by intracellular staining and CD107 expression by adding anti-CD107a+b antibodies during the restimulation. Dotplots are gated on tetramer^+^ CD8+ T cells. Representative of 8 experiments performed with 3 donors at day 8–40 of the culture. (B) MelA-specific T cells induced by the pDC line are cytotoxic. T cells were selected from the culture and submitted to a ^51^Cr release assay using peptide-loaded T2 cells and melanoma tumor cells as targets. Representative of 8 experiments performed with 4 donors at d13–40 of the culture. (C,D) IFNγ secretion and CD107 expression were assessed as described in (A) after three stimulations of PBMC and analyzed on the tetramer^+^ CD8+ T cells (white bars) and on the non-specific tetramer^-^ CD8+ T cells (grey bars) and CD4+ T cells (black bars) upon restimulation with peptide-pulsed T2 or GEN cells (4 experiments for each condition).

We further examined the functionality of the specific T cells induced by the HLA-A*0201 matched allogeneic pDC line. We first analyzed the ability of tumor-specific T cells to secrete IFNγ and express CD107 upon restimulation. When co-cultured with peptide-loaded T2 cells, MelA-specific T cells secreted IFNγ and expressed CD107 in the presence of the relevant but not control peptide (Figure 2A). We obtained within tumor-reactive T cells averages of 25% IFNγ^+^ tetramer^+^ CD8 T cells upon specific restimulation compared to 5% under control conditions (p = 0.007), and 50% CD107^+^ tetramer^+^ CD8 T cells upon specific restimulation compared to 24% under control conditions (p = 0.02) (data not shown). We next tested their cytotoxicity by performing ^51^Cr release assay using peptide-loaded T2 cells and melanoma tumor lines as targets. MelA-specific T cells exhibited a strong cytotoxic activity towards T2 cells loaded with the relevant but not with control peptide (Figure 2B). We obtained 88% of specific killing versus 13% under control conditions (mean of 8 experiments, p<0.001) (not shown). In addition, MelA-specific T cells were able to lyse HLA-A*0201^+^MelA^+^ (Me275) but neither HLA-A*0201^+^MelA^-^ (A375) nor HLA-A*0201^-^MelA^+^ (COLO829) melanoma tumor cells (Figure 2B, Figure S1) demonstrating the HLA-A*0201-restriction and antigen specificity of this activity. Furthermore, this lysis was inhibited by EGTA-MgCl2 or by CD8 T cell depletion (not shown), which together with CD107 surface expression upon specific restimulation, suggests a mechanism involving cytolytic granule exocytosis from CD8 T cells. Such functional capacities were observed with T cells taken at different timepoints of the 7–40 day culture. Similar analysis performed with virus-specific T cells demonstrated the capacity of Flu tetramer^+^ T cells to secrete IFNγ and express CD107 upon specific restimulation, and their cytotoxic properties (Figures S3A and S3B). Importantly, we observed only a minor allogeneic response induction as attested by the poor activation of non-specific tetramer^–^ CD8+ T cells and CD4+ T cells towards GEN cells. This was observed after one stimulation (Flu response) (Figure S3C and S3D) but also after repeated stimulations with the pDC line (MelA response), by measuring IFNγ-secreting (Figure 2C) and CD107-expressing T cells (Figure 2D) upon restimulation with T2 or GEN cells. We also observed the absence of the tetramer^+^ T cell activity towards GEN cells loaded with an irrelevant peptide. Thus, the pDC line elicits fully functional antigen-specific T cells with minor bystander allogeneic responses.

### The peptide-loaded HLA matched allogeneic pDC line elicits strong in vivo antigen-specific T cell responses in humanized mice

**Figure 3:**
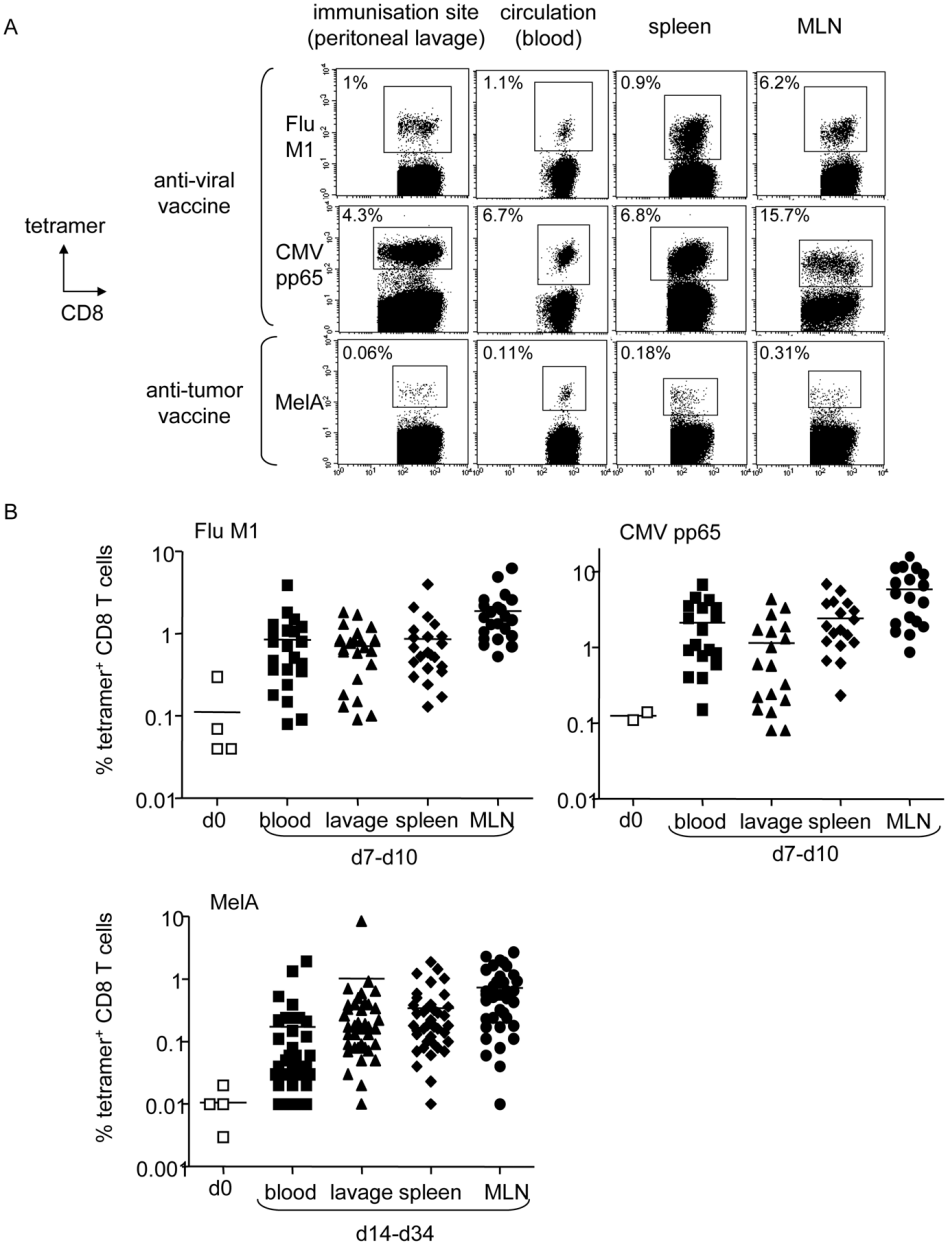
Vaccination with the peptide-loaded HLA-A*0201 matched allogeneic pDC line elicits strong antigen-specific T cell responses in humanized mice. (A-B) Immunodeficient NOD-SCIDβ_2_m^-/-^ mice were reconstituted intraperitoneally with 50.10^6^ human HLA-A*0201^+^ healthy donors' PBMC and vaccinated by the same route with 5.10^6^ irradiated peptide-loaded GEN cells. Specific T cell induction was analyzed at the injection site (lavage), in the circulation (blood) and lymphoid organs (spleen, LN) by tetramer labelling of human T cells in cell suspensions. (A) Vaccination with peptide-loaded GEN cells induced specific T cell responses in vivo. Representative dot plots of tetramer labeled T cells induced after a single vaccination with peptide-loaded GEN cells in different organs at day 8 for anti-viral vaccine (Flu, CMV) and day 10 for anti-tumor vaccine (MelA) (gated on CD8^+^ T cells). One mice per group is shown. Initial levels of specific T cells within PBMC were 0.04%, 0.14% and 0.003% respectively. (B) Levels of specific T cells before (day 0) and after vaccination with GEN loaded with FluM1 (n = 22 mice, 4 donors, 1 vaccine), CMVpp65 (n = 18 mice, 2 donors, 1 vaccine) and MelA (n = 38 mice, 4 donors, 2–3 vaccines) peptides at the indicated times in different organs. Each dot represents one vaccinated HuPBL mice (bars at mean).

The use of HLA matched allogeneic pDCs as a vaccine requires induction of antigen-specific T cell responses in vivo. Therefore we evaluated the vaccine potential of the pDC line in a humanized mouse model [42] constructed by xenotransplanting human PBMC into immunodeficient NOD-SCIDβ_2_m^-/-^ mice (HuPBL SCID model). Twenty four hours after intraperitoneal transfer, human CD45^+^ haematopoietic cells were found at the injection site but also in the circulation and lymphoid organs (not shown). A single intra-peritoneal injection of the irradiated peptide-loaded HLA-A*0201 matched allogeneic pDC line induced strong antigen-specific T cell responses towards viral (FluM1, CMVpp65) and tumor (MelA) antigens in HuPBL mice (Figure 3A). Human tetramer^+^ CD8 T cells were found not only at the site of immunization (peritoneal lavage) but also in the circulation (blood) and lymphoid organs (spleen, lymph nodes) (Figure 3A). We then evaluated whether several weekly injections of the peptide-pulsed pDC line could enhance the level of the response. Interestingly, viral antigen (Flu) induced a high response that peaked 7 days after the first vaccine and decreased afterwards, whereas response to tumor antigen (MelA) kept increasing upon subsequent restimulations (Figure S4). Within all vaccinated HuPBL mice (n = 22, 18 and 38) reconstituted with human PBMC (baseline levels of tetramer+ CD8+ T cells were 0.11% (FluM1), 0.12% (CMVpp65) and 0.01% (MelA) tetramer^+^ T cells) levels of specific T cells recovered in the different organs ranged from 0.7 to 1.9% for FluM1, 1.1 to 5.9% for CMVpp65, and 0.2 to 1% for MelA (Figure 3B). Thus, the peptide-pulsed pDC line elicits strong and widespread antigen-specific T cell responses in vivo.

### Vaccination with the peptide-loaded HLA matched allogeneic pDC line protect humanized mice from tumor progression in both prophylactic and therapeutic settings

**Figure 4:**
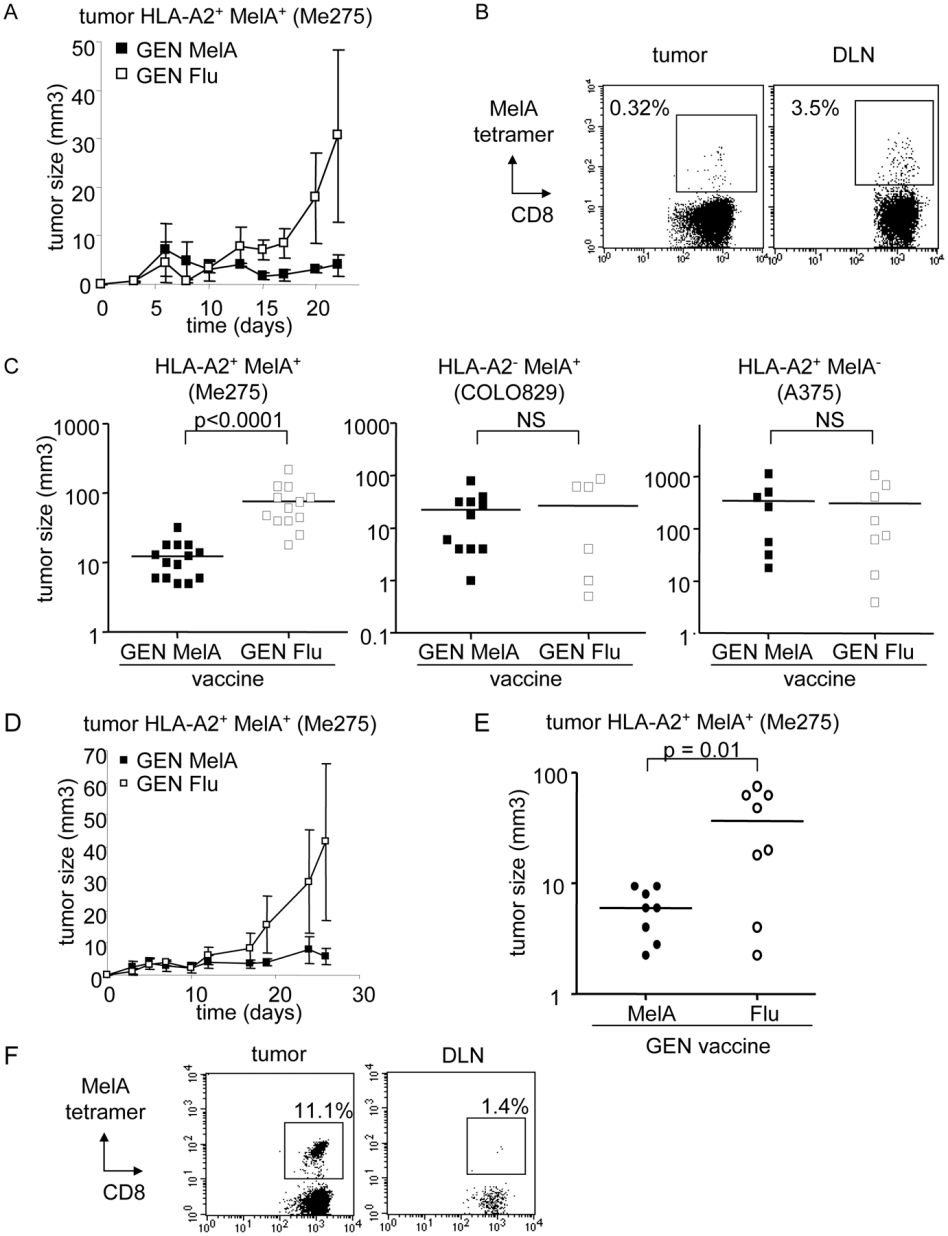
Vaccination with the peptide-loaded HLA-A*0201 matched allogeneic pDC line protect humanized mice from tumor development both prophylactically and therapeutically. (A-C) Immunodeficient NOD-SCID β_2_m^-/-^ mice reconstituted intraperitoneally with human HLA-A*0201^+^ PBMC (HuPBL mice) were weekly vaccinated subcutaneously with irradiated MelA or Flu-loaded GEN cells and challenged 5 days later with melanoma tumor cells in the flank. (A) Follow up of tumor progression. One experiment representative of 5. (B) Tetramer labelling of tumor and draining LN cell suspensions from HuPBL mice vaccinated with MelA-loaded GEN cells showing the presence of MelA-specific T cells (gated on CD8+ T cells). (C) The therapeutic effects of the vaccine are HLA-A*0201-restricted and antigen-specific. Comparative tumor size 27 days after implantation of Me275, COLO829 and A375 melanoma cells into HuPBL mice vaccinated with MelA or Flu-loaded GEN cells (pool of 3 independent experiments for each tumor type performed with 6 to 14 mice per group). (D-F) Immunodeficient NOD-SCID β_2_m^-/-^ mice reconstituted intraperitoneally with human HLA-A*0201^+^ PBMC (HuPBL mice) were first challenged with melanoma Me275 tumor cells in the flank and then vaccinated subcutaneously with irradiated MelA or Flu-loaded GEN cells weekly starting 4 days later. (D) Follow up of tumor progression. One representative experiment out of 2. (E) Comparative tumor size 25 days after tumor implantation (pool of 2 independent experiments, 8 mice/group). (F) Tetramer labelling of tumor and draining LN cell suspensions from HuPBL mice vaccinated with MelA-loaded GEN cells showing the presence of MelA-specific T cells (gated on CD8+ T cells).

We next investigated the therapeutic potential of this strategy in humanized mice further engrafted with human tumor. NOD-SCIDβ_2_m^-/-^ mice were reconstituted intra-peritoneally with human HLA-A*0201^+^ PBMC and weekly vaccinated subcutaneously with irradiated peptide-pulsed GEN cells before or after being challenged with melanoma tumor cells. In a prophylactic setting, injection of HuPBL mice with MelA-loaded GEN cells, compared to Flu-loaded GEN cells, inhibited HLA-A*0201^+^MelA^+^ tumor growth (Me275) in five independent experiments (tumor size at day 27 = 12 vs 77 mm^3^, p<0.0001) (Figure 4A and 4C). By contrast, the growth of HLA-A*0201^-^MelA^+^ (COLO829) and HLA-A*0201^+^MelA^-^ (A375) melanoma tumors was not affected after injection of MelA or Flu-loaded GEN cells in three independent experiments (Figure 4C), demonstrating the HLA-A*0201-restriction and antigen specificity of the therapy. Moreover, the peptide-loaded pDC also provoke protective immune responses against already established tumors. Vaccination of tumor-bearing HuPBL mice with MelA-loaded GEN cells inhibited tumor growth compared to Flu-loaded GEN cells (tumor size at day 25 = 6 vs 36mm^3^, p = 0.01) (Figure 4D-4F). Notably, tetramer^+^ CD8+ T cells were found at the tumor site and in the draining LN (Figures 4B and 4F), suggesting that the tumor-reactive T cells induced by the HLA matched allogeneic pDC had migrated to the site of antigen expression and the T cells were capable of killing tumor cells.

### The HLA matched allogeneic pDC line loaded with melanoma-derived peptides induces multi-specific and highly functional T cell responses ex-vivo from stage I-IV melanoma patients

**Figure 5:**
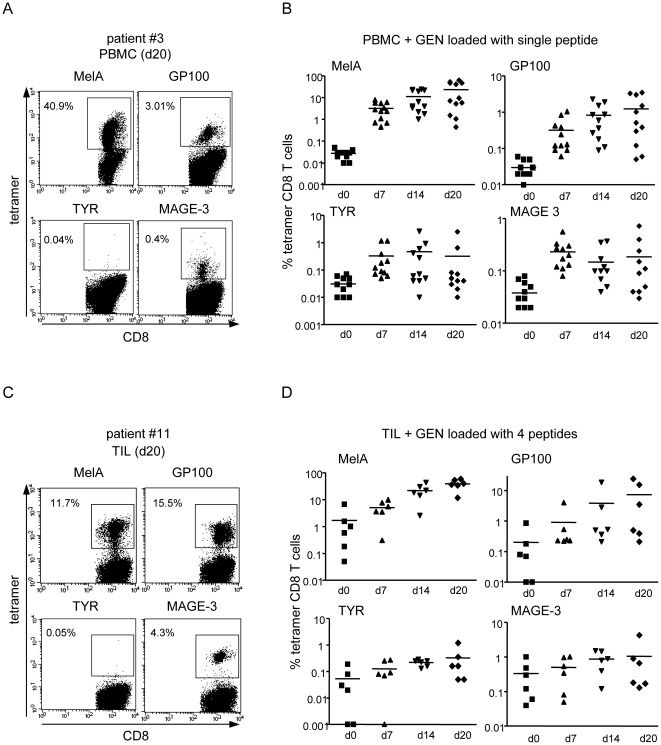
The HLA-A*0201 matched allogeneic pDC line loaded with melanoma-derived peptides induces multi-specific T cell responses ex-vivo from stage I-IV melanoma patients. PBMC (n = 12) and TIL (n = 6) obtained from stage I-IV HLA-A*0201^+^ melanoma patients were cultured with irradiated GEN cells loaded with MelA, GP100, TYR and/or MAGE-3 derived peptides and restimulated every 7 days. Percentages of specific T cells were determined by tetramer labelling after culture of PBMC with single peptide-loaded GEN cells (A,B) and of TIL with GEN loaded with a mix of the 4 peptides (C,D). Representative experiments with PBMC (A) and TIL (C) are shown at day 20 of the culture. Results from PBMC and TIL cohorts are shown in (B) and (D) at days 0, 7, 14 and 20 of culture. For TYR (D), one patient was excluded due to an extremely intense response (see Figure S5).

**Figure 6:**
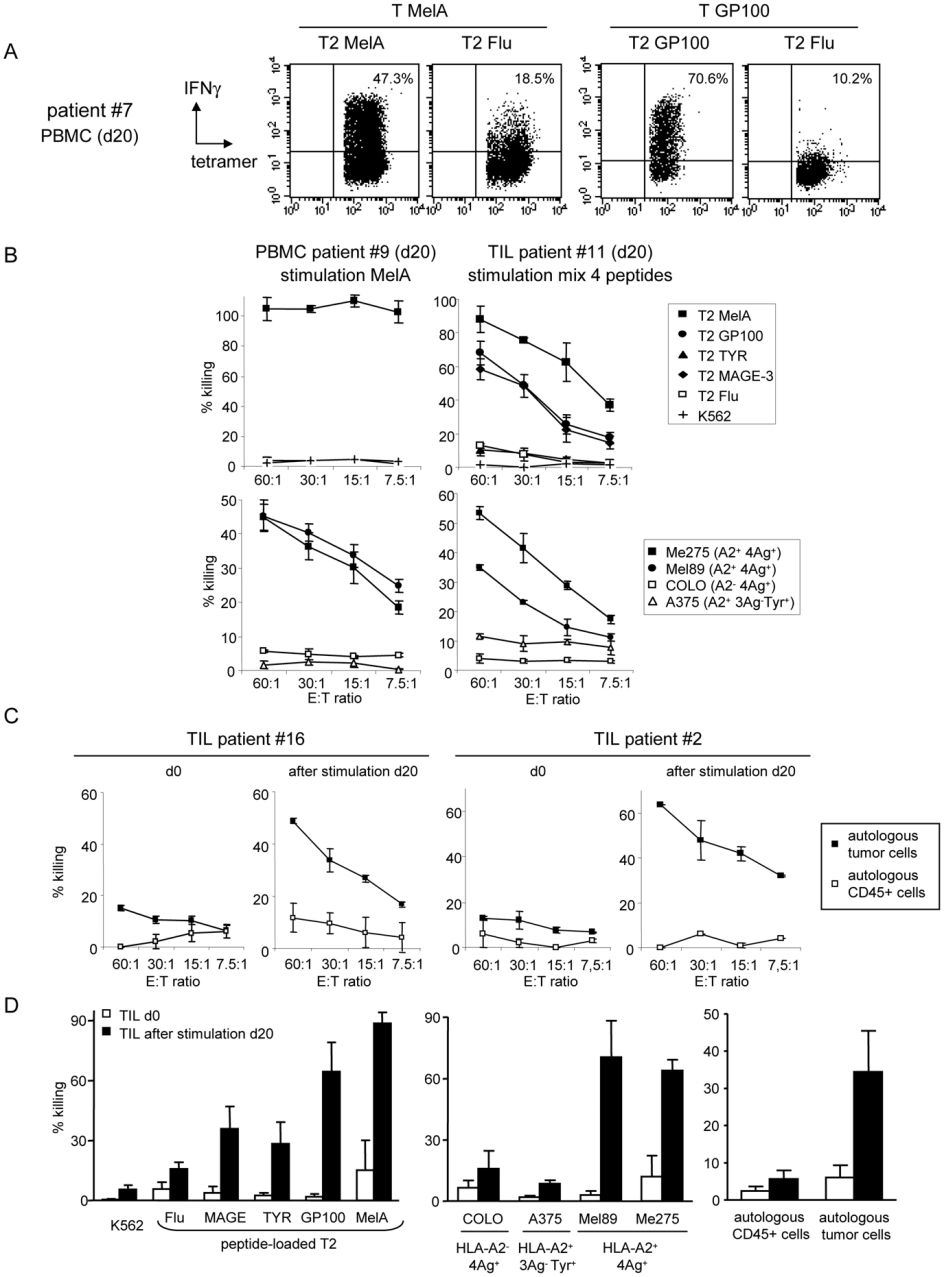
The melanoma patients' tumor-specific T cells induced by the HLA-A*0201 matched allogeneic pDC line are highly cytotoxic and lyse autologous melanoma tumor cells. Tumor-specific T cells induced by the pDC line loaded with melanoma-derived peptides from melanoma patients' PBMC and TIL are highly functional in an HLA-A*0201 and antigen-specific manner. (A) Tetramer^+^ T cells specifically secreted IFNγ upon restimulation with T2 cells pulsed with the relevant peptide. Representative of 6 PBMC and 2 TIL samples. (B-D) T cells exhibited cytotoxicity towards allogeneic and autologous melanoma tumor cells and relevant peptide-pulsed T2 cells. T cells induced from PBMC or TIL were purified from days 15–20 cultures and used in a ^51^Cr release assay against peptide-loaded T2 cells, allogeneic and autologous melanoma tumor cells, and autologous CD45^+^ cells. (B) The PBMC sample shown was stimulated with MelA-loaded GEN cells. The TIL sample shown was stimulated with GEN cells loaded with a mixture of 4 peptides and developed a response towards MelA, GP100 and MAGE-3 antigens (see Figure 5C). Representative of 12 PBMC and 6 TIL samples. (C) Percentage of killing of autologous tumor cells compared to autologous CD45^+^ cells by TIL before and after stimulation. Representative of 6 TIL samples. (D) Comparison of the killing capacity between unstimulated and stimulated TIL on the indicated targets at a 60∶1 ratio. Mean+/-SEM of 6 TIL samples.

We next investigated the relevance of this strategy in cancer patients. We tested the capacity of the peptide-pulsed pDC line to trigger ex-vivo tumor-specific responses from PBMC and tumor-infiltrating lymphocytes (TIL) isolated from stage I-V HLA-A*0201^+^ melanoma patients (Tables S1 and S2). Weekly stimulations of patients' PBMC with the pDC line pulsed either with MelA, GP100, TYR or MAGE-3 peptide led to the massive amplification of specific CD8+ T cells for at least 2 out of 4 melanoma antigens (Figures 5A and 5B). The tumor-specific tetramer^+^ CD8 T cell responses reached averages of 23% for MelA (range 0.4–62%), 1.2% for GP100 (range 0.05–3.5%), 0.3% for TYR (range 0.01–2.5%) and 0.2% for MAGE-3 (range 0.03–0.72%) after 20 days (baseline tetramer+ CD8+ T cells ranged from 0.02 to 0.03% at d0). One patient was excluded from the analysis due to an extremely intense response (85% tetramer^+^ T cells at day 14) towards TYR (Figure S5). Furthermore, repeated stimulations of patients'TIL with the pDC line pulsed with a mix of the 4 melanoma peptides led to the massive amplification of specific T cells for at least 3 antigens (Figures 5C and 5D). Tumor-specific tetramer^+^ CD8 T cell responses reached averages of 39% for MelA (range 12–59%), 7.4% for GP100 (range 0.2–24%), 0.3% for TYR (range 0.05–1.2%) and 1.1% for MAGE-3 (range 0.1–4.3%) after 20 days with baseline levels format day 0 1.7, 0.2, 0.05 and 0.3%, respectively. The tumor-specific T cells induced from both PBMC (Figure 6A) and TIL (not shown) secreted IFNγ when co-cultured with T2 cells loaded with the relevant but not with a control peptide. We obtained a mean of 42% of IFNγ^+^ tetramer^+^ CD8 T cells upon specific peptide restimulation compared to 11% in control conditions (n = 12, p = 0.0002, data not shown). Furthermore, these T cells exhibited a strong cytotoxic activity towards T2 cells loaded with relevant but not with control peptides and allogeneic melanoma tumor cells in an HLA-A*0201-restricted and antigen-specific manner (Figure 6B). Strikingly, after stimulation with the pDC line loaded with a mix of four melanoma-derived peptides, TIL acquired the ability to lyse autologous tumor cells but not CD45^+^ hematopoietic cells from the patient (Figure 6C). When comparing the killing capacity of unstimulated and stimulated TIL, the pDC line greatly enhanced their cytotoxic activity towards peptide-loaded T2 cells, semi-allogeneic melanoma tumor lines and autologous tumor cells (Figure 6D). Thus, the HLA- matched allogeneic pDC line loaded with melanoma-derived peptides induces multi-specific and highly functional ex-vivo T cell responses from stage I-IV melanoma patients.

### The HLA matched allogeneic pDCs are more potent at inducing tumor-specific T cells than conventional autologous or HLA matched allogeneic mDCs

**Figure 7:**
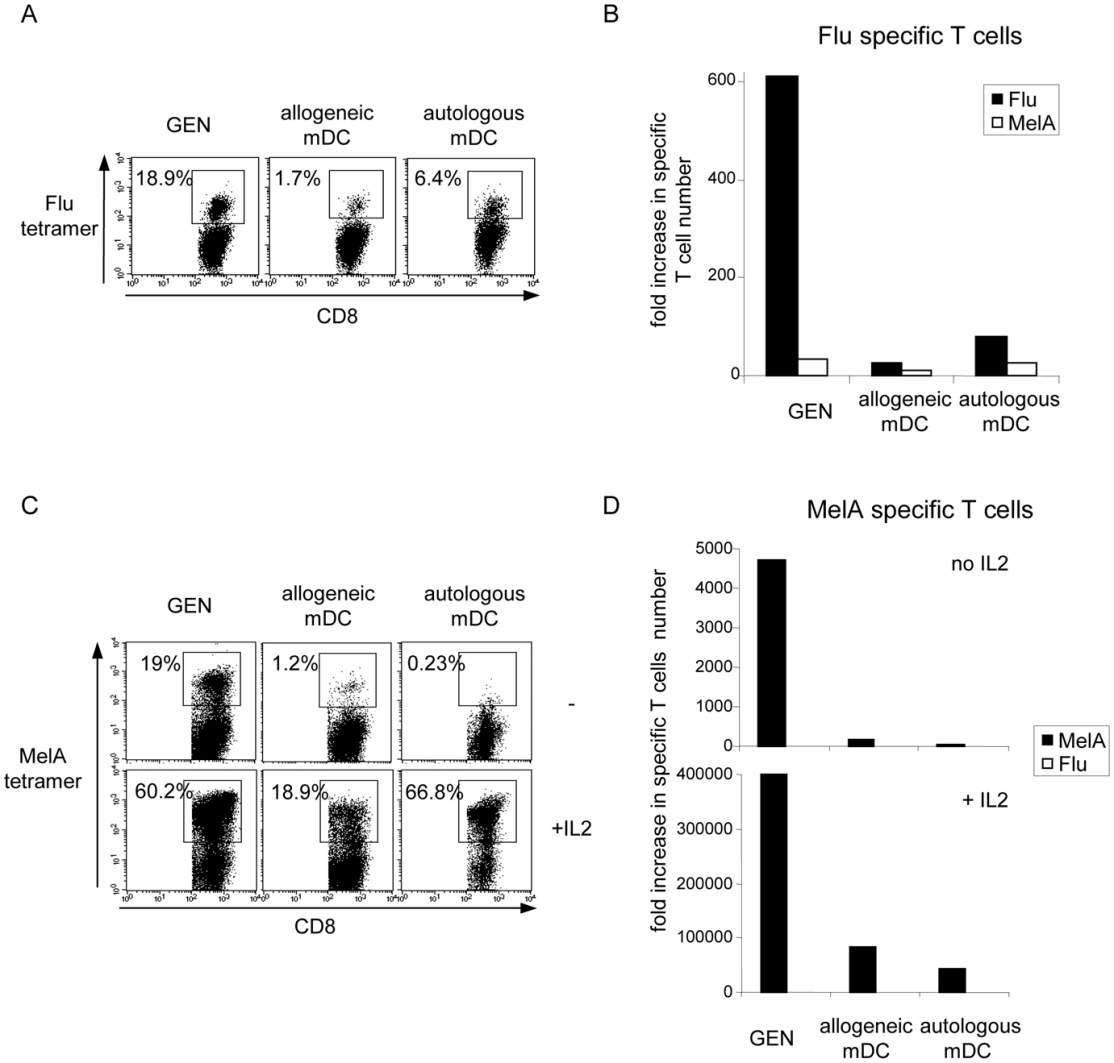
The HLA-A*0201 matched allogeneic pDC line is more effective than conventional mDCs. PBMC from HLA-A*0201^+^ healthy donors were stimulated either with the pDC line or with allogeneic or autologous HLA-A*0201^+^ mDCs loaded with Flu (A, B) or MelA (C, D) peptide in presence of IL-2 where indicated. (A) Percentages of FluM1 tetramer^+^ CD8+ T cells and (B) fold increase in specific T cell number at d7 of culture. MelA tetramer was used as control. (C) Percentages of MelA tetramer^+^ CD8+ T cells and (D) fold increase in specific T cell number at d20 of culture. Flu tetramer was used as control.

**Figure 8:**
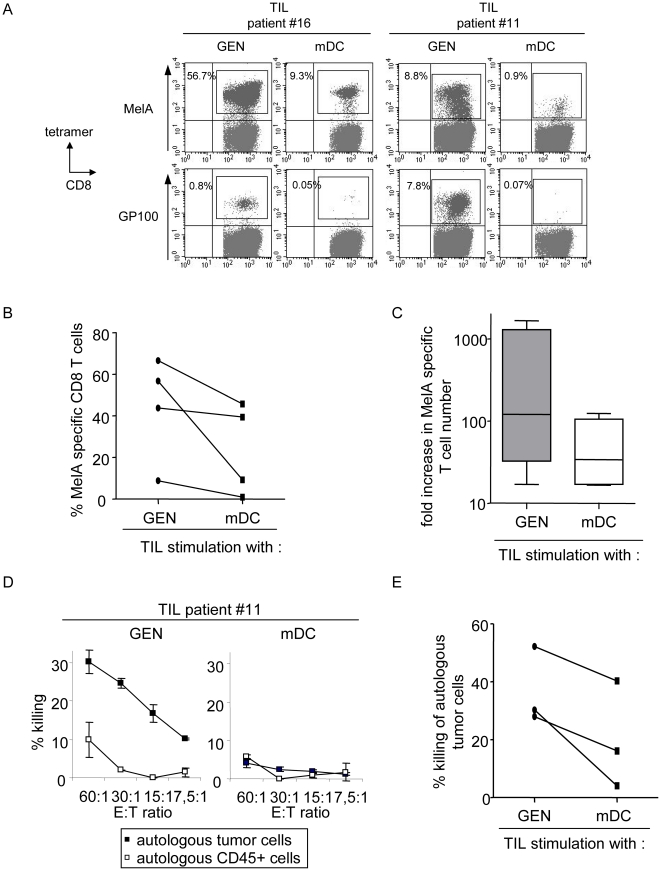
The HLA-A*0201 matched allogeneic pDCs are more potent at inducing tumor-specific T cells from melanoma patients than conventional mDCs. TIL from melanoma patients were cultured either with irradiated GEN cells or allogeneic LPS-matured HLA-A*0201^+^ mDCs loaded with a mixture of MelA, GP100, TYR and MAGE-3 derived peptides. Percentages of tumor-specific T cells were determined by tetramer labelling after three stimulations at day 20 of culture. (A) Representative dotplots of two cultures. (B) Comparative percentages of MelA-specific T cells and (C) fold increase in specific T cell numbers upon stimulation of TIL with GEN or mDCs (4 patients). (D,E) T cells were purified from the culture and submitted to a ^51^Cr release assay. (D) Percentage of killing of autologous tumor cells and autologous CD45^+^ cells after TIL stimulation with GEN or mDCs. One representative patient is shown. (E) Comparative killing efficacy against autologous tumor cells at a 60∶1 ratio.

We next compared the T cell stimulatory capacity of the pDC line against conventional mDCs in autologous and semi-allogeneic settings. HLA-A*0201^+^ PBMC from healthy donors were stimulated with irradiated peptide-loaded GEN cells, HLA-A*0201^+^ allogeneic mDCs or autologous mDCs. The virus-specific (FluM1) and tumor-specific (MelA) responses elicited respectively after a single or three stimulations were higher when using GEN cells compared to allogeneic or autologous mDCs (Figure 7A-7D). Similarly, stimulation of TIL from melanoma patients with GEN cells led to a more potent induction of tumor-specific T cells than semi-allogeneic mDCs in respect to both tetramer+ T cell percentage (Figures 8A and 8B) and fold increase in absolute number of specific T cells (Figure 8C). Notably, the pDC line elicited specific T cells with better functional qualities, as assessed by more potent CTL activity towards autologous tumor cells from melanoma patients, compared to the T cells induced by conventional HLA matched allogeneic mDCs (Figures 8D and 8E). Thus, the induction of specific T cell responses by the pDC line is much more potent compared to that elicited by autologous or HLA matched allogeneic myeloid DCs.

In order to investigate the mechanism of the high efficiency of the pDC line, we analysed changes in the activation level of the pDCs. The 30Gy irradiation provided to the pDC line induced a strong activation of the cells as demonstrated by the upregulation of costimulatory molecules (CD40, CD80, CD86), upregulation of MHC-I molecules (Figure S6). In addition, by using IFNαβ blocking antibodies, we observed that the induction of specific T cell responses by the pDC line was not abrogated, suggesting a type I IFN independent mechanism (Figure S7). We also addressed the comparative avidity of the specific T cells elicited by the pDC line or mDCs by measuring their cytotoxic activity upon stimulation with titrated peptide-MHC complexes. We observed that the specific T cells elicited by the pDCs kept the same level of functionality towards T2 target cells loaded with up to 0.01 µM whereas the specific T cells elicited by allogeneic mDCs displayed a 50% reduced functionality at this peptide concentration (Figure S8). Therefore, the pDCs elicited high avidity specific T cells.

## Discussion

pDCs can act as antigen presenting cells and they have been demonstrated to play a role in tumor-specific responses [43]. However, the potential for pDCs in clinical application has not yet been worked out. In this study, we explored the feasibility of peptide-loaded HLA matched allogeneic pDC to induce tumor immunity. We demonstrate here the strong potential of this strategy in inducing multi-specific and highly functional CD8+ T cell responses from healthy donors as well as cancer patients. These findings provide a pre-clinical basis for the use of HLA matched allogeneic pDCs as vectors for cancer immunotherapy.

The use of pDCs to induce T cell responses has already been investigated in vitro and in mouse models of cancer and infection [21], [31], but its further development for clinical application was impractical due to the absence of an easy way to generate or purify human pDCs in sufficient number. As peptide-pulsed primary pDCs isolated from the blood induced higher T cell responses in HLA matched allogeneic settings compared to autologous conditions, we further investigated the potential of pDC combined with HLA matched allogeneicity using the HLA-A*0201^+^ pDC line. We demonstrated that the irradiated peptide-pulsed pDC line can strongly trigger both primary and memory specific T cell responses more effectively than conventional myeloid DCs. The use of pDC in an allogeneic context with a partial HLA matching between the vaccine and the responding T cells resulted in an enhancement of HLA-A*0201-restricted specific responses, with only minor allogeneic response induction as assessed by the low level of non-specific T cell activation upon restimulation with the vaccine. Thus, there is a strong enhancement induced by HLA matched allogeneicity [37], that is further potentiated by a pDC vaccineThe safety of allogeneic cell therapies has already been assessed in cancer clinical trials [44]–[46]. Several trials demonstrated in cancer patients that the use of HLA matched allogeneic mDCs or mDCs/tumor hybrids induced strong anti-tumor immune responses and clinical responses [47], [48]. These studies clearly demonstrate the feasibility of generation of tumor-specific T cell responses in vivo on an allo-background. HLA-restricted tumor specific T cell responses are not drowned out by bystander alloresponses.

The use of HLA-matched allogeneic pDCs may explained the relative important HLA-restricted responses over alloresponses induction, and its efficacy. It has been shown that pDCs can induce allogeneic T cell hyporesponsiveness and subsequent prolonged graft survival [49]; this may be due to a high ratio of B7-H1 to B7-1 and B7-2 molecule expression that in turn influence the outcome of their interactions with T cells. It has also been suggested that pDCs are equipped with large “ready-made” intracellular stores of MHC-I molecules than can be rapidly mobilize to the cell surface to initiate antigen-specific CD8 T cell responses [50]. The localization of MHC-I molecules in the recycling endosomal compartment suggests a rapid translocation to the cell surface after stimulation. In addition pDCs cross-present antigens more rapidly and efficiently than do mDCs and this process is IFNα-independent. In line with this, we observed a rapid upregulation of MHC-I molecules on the peptide-loaded pDC line upon activation and the induction of specific T cells was IFNα-independent (not shown). These differences of MHC-I molecules mobilization at early time points between pDCs and mDCs may explain the superiority of pDCs over mDCs. In addition, pDCs and mDCs handle MHC-II molecules differently after activation [51]: pDCs continuously turn over MHC-II molecules after activation whereas mDCs did not which results in sustained surface expression of individual peptide-MHC-II molecules complexes. pDCs display low amounts of MHC-II molecules on their surface and did not upregulate MHC-II synthesis soon after activation [52]. A possible explanation for the selective HLA restricted- over allo-responses triggering by pDCs could be the differential mobilization of MHC-I and MHC-II molecules upon stimulation and the rapid mobilization of MHC-I molecules to the cell surface. We observed that the specific T cell induction by the pDCs was type I IFN independent (not shown). Furthermore, the irradiation of the pDC line induced a strong cell activation as assessed by the upregulation of the costimulatory markers CD40, CD80, CD86 and HLA-A2 molecule and the secretion of pro-inflammatory cytokines (not shown) to similar levels to the one provided by TLR-7 or TLR-9 stimulation. On the contrary mDCs did not upregulate these costimulatory molecules upon irradiation. These unique features of pDCs could allow a preferential MHC-I orientation leading to HLA restricted- over allo-responses induction and contribute to its high HLA-A*0201 restricted CD8 T cell stimulatory capacity.

Tumor-specific T cells induced by the HLA matched allogeneic pDC were highly functional as demonstrated by the capacity of tetramer^+^ T cells to secrete IFNγ and express CD107 upon specific restimulation, and their strong antigen and HLA-A*0201-specific cytotoxicity. As peptide-loaded T2 cells are more susceptible to lysis than cells expressing the antigen endogenously, we performed each cytotoxicity assay not only on peptide-loaded T2 cells, but also on several allogeneic melanoma cell lines and importantly on autologous melanoma tumor cells from the patients themselves. Our results demonstrate that the specific T cells induced by the pDC line are able to kill tumor cells expressing the antigen endogenously. The phenotype of the effector T cells generated was that of intermediate effectors: CD27+ CD45RO+ CD62L- T cells (not shown), suggesting that they were not terminally differentiated even after serial stimulations. Importantly, intermediate effector cells have been indicated to be optimal for in vivo efficacy [53]. We also observed that the specific T cells elicited by the pDCs had a better affinity and higher avidity compared to specific T cells triggered by allogeneic mDCs, as suggested by a slowest tetramer dissociation rate and a better functionality on titrated peptide-MHC complexes (not shown). These observations suggest that the specific T cells elicited by the pDC vaccine will be able to recognize antigen at the physiological low concentrations. We also demonstrated the strong potential of the peptide-loaded pDC line to induce specific T cell responses in a humanized mouse model, and its efficacy in inhibiting the development of already established tumors.

Importantly, we further provide clinical relevance of this strategy by demonstrating it potently stimulates highly functional tumor-specific T cells from melanoma patients. After stimulation with the pDC line loaded with a mix of four melanoma peptides, T cells from melanoma patients acquired the ability to kill autologous tumor cells. Such results were obtained with PBMC as well as with TIL taken from all tested patients, at different stages of their disease and regardless of their previous treatment, providing the preclinical evidence for the strong potential of our strategy.

We performed a comparison of our strategy with frequently used myeloid derived dendritic cells. As demonstrated for several antigens and with healthy donors' as well as melanoma patients' cells, the pDC line led to a more potent induction of tumor-specific T cells endowed with better functional capacities compared to conventional mDCs. CTL generated by the pDC line are much more effective in killing autologous melanoma tumor cells compared to CTL generated by mDC. Allogeneic leukaemic myeloid cell lines that have DC properties have also been evaluated for their potential to prime tumor-specific CTL [54], [55], but our strategy is superior in many aspects including the cell line generation, the settings required for induction of T cell responses, and the efficacy of specific T cells elicited.

The efficacy and design of this immunotherapeutic approach render the strategy very attractive for further clinical developments. Our peptide-pulsed pDC line can be provided directly as a ready-to-use GMP secured frozen vaccine. Notably, it represents a multi-usage flexible strategy, as the same vaccine can be used for every HLA-A*0201^+^ patient, and by utilizing target antigens it can be suitable for various pathologies. Regarding clinical use, we have already addressed a number of biosafety issues, in accordance with regulatory requirements.

This work demonstrates that HLA matched allogeneic pDCs are promising vectors for cancer immunotherapy. GENiusVac represents a real advancement in the challenging area of cancer immunotherapy with broad clinical applications.

## Supporting Information

Figure S1 HLA-A2 and tumor antigen expression by melanoma tumor cell lines. (A) Analysis by flow cytometry of surface HLA-A2 (top panels) and intracellular MelA (lower panels) expression by Me275, Mel89, COLO829 and A375 melanoma tumor cell lines. (B) Analysis by real time PCR of MelA, GP100, tyrosinase and MAGE-3 gene expression by Me275, Mel89, COLO829 and A375 melanoma tumor cell lines.
(4.76 MB TIF)

Figure S2 The HLA-A*0201 matched allogeneic pDC line induces highly effective virus-specific T cell responses from HLA-A*0201^+^ healthy donors' PBMC in vitro. Allogeneic HLA-A*0201 PBMC from healthy donors were stimulated with the irradiated peptide-loaded HLA-A*0201^+^ pDC line. Specificity of the T cells was determined by tetramer labelling and flow cytometric analysis. (A) Representative dot plot of Flu M1 tetramer labelling at d0 and d7 of culture (gated on CD8+ T cells). (B) Percentages of Flu M1 tetramer^+^ CD8+ T cells determined at d0 and d7 of culture (n = 20). (C) Representative dot plot of CMVpp65 tetramer labelling at d0 and d7 of culture (gated on CD8+ T cells). (D) Percentages of CMV pp65 tetramer^+^ CD8+ T cells determined at d0 and d7 of culture (n = 14). Anti-viral tetramer^+^ T cell responses reached averages of 11% for FluM1 (range 0.1–49) and 12% for CMVpp65 (range 0.04–76) in 7 days starting from respectively 0.23 and 0.18%.
(5.25 MB TIF)

Figure S3 The virus-specific T cells induced in vitro by the HLA-A*0201 matched allogeneic pDC line exhibited functional HLA-A*0201- and antigen-specific activity. (A) Flu-specific T cells induced by the pDC line secrete IFNγ and express CD107 on the surface upon restimulation. Cells from the culture (day 8) were tetramer labeled and restimulated with T2 cells pulsed with a relevant or control peptide. IFNγ production was assessed by intracellular staining and CD107 expression by adding anti-CD107a+b antibodies during the restimulation. Dotplots are gated on tetramer^+^ CD8+ T cells. Representative of 4 experiments performed with 3 donors at day 8-10 of the culture. (B) Flu-specific T cells induced by the pDC line are cytotoxic. T cells were selected from the culture and submitted to a ^51^Cr release assay using peptide-loaded T2 cells as targets. T cells killed T2 cells loaded with the relevant but not the control peptide. Representative of 7 experiments performed with 4 donors at d7-10 of the culture. (C,D) IFNγ secretion and CD107 expression were assessed as described in (A) after a single stimulation of PBMC and analyzed on the tetramer^+^ CD8+ T cells (white bars) and on the non-specific tetramer^-^ CD8+ T cells (grey bars) and CD4+ T cells (black bars) upon restimulation with peptide-pulsed T2 or GEN cells (4 experiments for each condition).
(4.90 MB TIF)

Figure S4 Response kinetics to vaccination with peptide-loaded GEN cells in humanized mice. Immunodeficient NOD-SCID β_2_m^-/-^ mice were reconstituted intraperitoneally with 50.10^6^ human HLA-A*0201 PBMC and weekly vaccinated by the same route with 5.10^6^ irradiated peptide-loaded GEN cells. Specific T cell induction was analyzed at different timepoints after vaccination at the injection site (lavage), in the circulation (blood) and lymphoid organs (spleen, MLN) by tetramer labelling of human T cells in cell suspensions. (A) Tumor-specific response to MelA-loaded GEN vaccination and (B) virus-specific response to Flu-loaded GEN vaccination. Each dot represents one vaccinated HuPBL mice.
(4.64 MB TIF)

Figure S5 TYR-specific T cells induction. Percentage of TYR-specific T cells determined at different timepoints of the PBMC culture from a melanoma patient (#4) with TYR-loaded GEN cells (gated on CD8+ T cells).
(4.49 MB TIF)

Figure S6 The 30Gy irradiation induced activation of the pDC line. pDCs were either untreated or submitted to a 30Gy irradiation. Twenty four hours later, the expression of costimulatory and HLA-A2 molecules were analysed by flow cytometry (representative of three experiments).
(4.38 MB TIF)

Figure S7 The induction of specific T cells by the pDC line is type I IFN independent. Allogeneic HLA-A*0201^+^ PBMC from healthy donors were stimulated with the irradiated Flu- or MelA-peptide loaded HLA-A*0201^+^ pDC line in the presence of anti-IFNα and anti-IFNβ antibodies or control goat IgG. The level of specific T cells was determined at day 7 of the culture by tetramer labelling and flow cytometry analysis.
(4.21 MB TIF)

Figure S8 Comparative avidity of the specific T cells elicited by the pDC line and allogeneic mDCs. Allogeneic HLA-A*0201^+^ PBMC from healthy donors were stimulated with the irradiated Flu-peptide loaded HLA-A*0201^+^ pDC line or HLA-A*0201^+^ mDC. At day 7 of the culture, the cytotoxic activity of the specific T cells was evaluated by a ^51^Cr release assay on T2 cells loaded with decreasing concentrations of Flu peptide (1 μM to 0.0001 μM), irrelevant peptide or unloaded. Data are expressed as a percentage of the maximum lysis measured for a E:T ratio of 30:1.
(4.21 MB TIF)

Table S1 Melanoma patients' samples and clinical parameters description.
(4.57 MB TIF)

Table S2 Initial levels of tetramer^+^ CD8 T cells within melanoma patients' samples.
(4.57 MB TIF)
